# Supplementary material for: Social Support’s Dual Mechanisms in the Loneliness–Frailty Link Among Older Adults with Diabetes in Beijing: A Cross-Sectional Study of Mediation and Moderation
Source: Healthcare (Basel). 2025 Jul 16;13(14):1713. doi: 10.3390/healthcare13141713 (PMC12294273; doi:10.3390/healthcare13141713)
Supplement: Supplementary file 1 [file healthcare-13-01713-s001.zip › healthcare-3666412-supplementary.pdf]

STROBE Statement—checklist of items that should be included in reports of observational studies

	Item No	Recommendation	Page/Paragraph in Manuscript
**Title and abstract**	1	(*a*) Indicate study design in title/abstract	Title: "A Cross-Sectional Study..." Abstract: "Methods: A cross-sectional study..."
		(*b*) Informative and balanced abstract summary	Abstract: Clear summary of background, methods, results, and conclusions.
**Introduction**			
Background/rationale	2	Scientific background and rationale	Introduction: Detailed context on aging, diabetes, loneliness, frailty, and gaps in research (China-specific challenges, theoretical frameworks).
Objectives	3	Specific objectives and hypotheses	Introduction: Explicitly states 3 objectives (quantify links, validate dual mechanisms, identify high-risk clusters). RQ1/RQ2 defined in Methods (2.1).
**Methods**			
Study design	4	Present key elements early	Abstract ("cross-sectional"), Introduction (final paragraph), Methods (2.1: "This cross-sectional study").
Setting	5	Setting, locations, dates, periods	Methods (2.1): Beijing, China; December 2024 - February 2025; recruitment period described.
Participants	6	Eligibility, sources, selection methods	Methods (2.1): Inclusion (≥60 yrs, T2D), exclusion criteria (cognitive, terminal illness, stressors), multistage stratified sampling protocol detailed.
Variables	7	Define outcomes, exposures, predictors, confounders, modifiers. Diagnostic criteria.	Methods (2.2): Clearly defines loneliness (UCLA), frailty (TFI ≥5), social support (SSRS). Covariates listed (age, gender, etc.). T2D criteria specified.
Data sources/ measurement	8*	Sources/details of measurement. Comparability.	Methods (2.2): Instruments named and described (UCLA, TFI, SSRS), validation cited (Cronbach's α, prior validation studies). Triangulation methods described.
Bias	9	Efforts to address bias	Methods (2.2): Triangulation to minimize recall bias (records, lab reports, anchor questions). Trained interviewers.
Study size	10	How study size was arrived at	Methods (2.1): Sample size calculation

			using G*Power (α=0.05, power=0.95, f²=0.15, 8 predictors, n=160 min). Accounted for non-response/stratification.
Quantitative variables	11	How handled in analyses. Grouping choices/why.	Methods (2.3): Preprocessing (missing data imputed via MICE, outliers Winsorized). Variables used continuously and categorically (e.g., SSRS tiers defined).
Statistical methods	12	(*a*) All statistical methods, confounding control	Methods (2.3): Pearson correlations, hierarchical linear regression (adjusting for 8 covariates), PROCESS macro (mediation Model 4, moderation Model 1).
		(*b*) Subgroup and interaction methods	Methods (2.3): Subgroup analysis (a priori high-risk categories), cluster analysis (k-means). Moderation tested via interaction term.
		(c) How missing data were addressed	Methods (2.3): "<5% imputed via MICE package".
		(d) Cross-sectional: Analytical methods accounting for sampling	Methods (2.3): Analyses account for stratification (covariates included).
		(e) Sensitivity analyses	Methods (2.3):Tested with 5,000 bootstrap samples.

Continued on next page

**Results**			
Participants	13*	(a) Numbers at each stage	Methods (2.1): 452 eligible identified → 5 excluded (cognitive) + 3 (terminal) + 2 (stressors) → 442 analysed (97.8% response).
		(b) Reasons for non-participation	Reasons for non-participation
		(c) Consider use of a flow diagram	The manuscript text describes the participant flow clearly (452 eligible -> 10 excluded -> 442 analyzed)
Descriptive data	14*	(a) Characteristics of participants, exposures, confounders	Results (3.1, Table 1, Table 2): Demographics, frailty/loneliness/support prevalence, scores, and distributions by subgroups.
		(b)Number with missing data per variable	Methods (2.3): "<5% imputed via MICE". Specific counts per variable not provided.
Outcome data	15*	Cross-sectional: Outcome events/summary measures	Results (3.1, Table 1): Frailty prevalence (55.2%), mean scores for frailty, loneliness, social support.
Main results	16	(a) Unadjusted & adjusted estimates, precision, confounders adjusted for	Results (3.2, Table 3: Correlations; 3.3, Table 4: Multivariable Regression β, p-values, CI; 3.4: Mediation effect & CI; 3.5, Table 5: Moderation β, p). Covariates listed (Table 4).
		(b) Category boundaries for continuous variables	Methods (2.2): Cutoffs defined for Loneliness (UCLA: Low 20-34, Mod 35-49, High 50-80), Social Support (SSRS: Low ≤22, Mod 23-44, High 45-66), Frailty (TFI ≥5).
		(c) Translate relative risk to absolute risk	Not applicable - primary analyses used correlations/regression, not RR.
Other analyses	17	Report other analyses (subgroups, interactions, sensitivity)	Results (3.6, Table 6: Subgroup analysis; 3.7, Table 7: Cluster analysis). Moderation is an interaction analysis. Sensitivity analyses not mentioned.
**Discussion**			
Key results	18	Summarise key results with reference to study objectives	Discussion (1st paragraph & 4.1): Summarizes high frailty prevalence, dual mechanisms (mediation 30.86%, moderation threshold SSRS≥45.47), high-risk clusters.
Limitations	19	Discuss limitations, sources of bias/imprecision, direction/magnitude of bias	Discussion (4.4): Explicitly notes cross-sectional design (no causality), urban sampling (generalizability), bidirectional plausibility, lack of

			inflammatory biomarker measurement.
Interpretation	20	Cautious overall interpretation considering objectives, limitations, other evidence	Discussion & Conclusions: Interprets findings cautiously, acknowledges bidirectionality, integrates findings with existing theory (Hawkley & Cacioppo, Cohen, Fried), discusses implications.
Generalisability	21	Discuss generalisability (external validity)	Discussion (4.4): Notes limitation of Beijing urban sampling, suggests need for validation in other regions/cultures. Conclusions mention "urban China".
**Other information**			
Funding	22	Source of funding and role of funders	Funding: "This research received no external funding".

*Give information separately for cases and controls in case-control studies and, if applicable, for exposed and unexposed groups in cohort and cross-sectional studies.

Note: An Explanation and Elaboration article discusses each checklist item and gives methodological background and published examples of transparent reporting. The STROBE checklist is best used in conjunction with this article (freely available on the Web sites of PLoS Medicine at http://www.plosmedicine.org/, Annals of Internal Medicine at http://www.annals.org/, and Epidemiology at http://www.epidem.com/). Information on the STROBE Initiative is available at www.strobe-statement.org.
